# A machine-learning approach to estimating public intentions to become a living kidney donor in England: Evidence from repeated cross-sectional survey data

**DOI:** 10.3389/fpubh.2022.1052338

**Published:** 2023-01-04

**Authors:** Paul Boadu, Leah McLaughlin, Mustafa Al-Haboubi, Jennifer Bostock, Jane Noyes, Stephen O'Neill, Nicholas Mays

**Affiliations:** ^1^Policy Innovation and Evaluation Research Unit, Department of Health Services Research and Policy, London School of Hygiene and Tropical Medicine, London, United Kingdom; ^2^School of Medical and Health Sciences, Bangor University, Bangor, United Kingdom

**Keywords:** public perceptions, public support, public intentions, living donor, cost-effectiveness, kidney donation, organ donation

## Abstract

**Background:**

Living kidney organ donors offer a cost-effective alternative to deceased organ donation. They enable patients with life-threatening conditions to receive grafts that would otherwise not be available, thereby creating space for other patients waiting for organs and contributing to reducing overall waiting times for organs. There is an emerging consensus that an increase in living donation could contribute even more than deceased donation to reducing inequalities in organ donation between different population sub-groups in England. Increasing living donation is thus a priority for National Health Service Blood and Transplant (NHSBT) in the United Kingdom.

**Methods:**

Using the random forest model, a machine learning (ML) approach, this study analyzed eight waves of repeated cross-sectional survey data collected from 2017 to 2021 (*n* = 14,278) as part of the organ donation attitudinal tracker survey commissioned by NHSBT in England to identify and help predict key factors that inform public intentions to become living donors.

**Results:**

Overall, around 58.8% of the population would consider donating their kidney to a family member (50.5%), a friend (28%) or an unknown person (13.2%). The ML algorithm identified important factors that influence intentions to become a living kidney donor. They include, in reducing order of importance, support for organ donation, awareness of organ donation publicity campaigns, gender, age, occupation, religion, number of children in the household, and ethnic origin. Support for organ donation, awareness of public campaigns, and being younger were all positively associated with predicted propensity for living donation. The variable importance scores show that ethnic origin and religion were less important than the other variables in predicting living donor intention.

**Conclusion:**

Factors influencing intentions to become a living donor are complex and highly individual in nature. Machine learning methods that allow for complex interactions between characteristics can be helpful in explaining these decisions. This work has identified important factors and subgroups that have higher propensity for living donation. Interventions should target both potential live donors and recipients. Research is needed to explore the extent to which these preferences are malleable to better understand what works and in which contexts to increase live organ donation.

## 1. Introduction

In developed countries with well-established healthcare systems living donation is common, offered as a routine part of healthcare, and proactively promoted to the public *via* media campaigns (1–4). As medical science and technology advances so does the scope of what is possible to retrieve from a living donor (5). Generally, in high income countries routine living donation will include blood (including cord blood) and plasma (1–4, 6, 7); breast milk (8); sperms and embryos (9); bone, tissue (including amniotic membrane, and the most common and more well-known liver lobe and kidneys (1–4). Globally, 31.5% of kidney transplants and 24.4% of liver transplants in 2020 were from living donors (10).

In the United Kingdom (UK) – a health service with a globally recognized live kidney donor programme (1–4)– a total of 2,567 kidney transplants occurred in 2020 and about 21.7% were from living donors (10). Generally, there are two pathways to become a living kidney donor: 1. donating to someone known to the donor e.g., a relative or friend, or 2. Altruistic (non-designated) donation. Altruistic (non-designated) donation can be directed, that is, donating to someone the donor has no prior relationship with but may be aware (normally *via* social media or a campaign from the potential recipient) of the need for a kidney donation, or non-directed, that is, a person agrees to donate a kidney but does not know, and will likely never know the recipient (1–4).

In the UK, the Living Kidney Sharing Scheme (UKLKSS) operated by National Health Service Blood and Transplant (NHSBT) ensures the best match between live donors and recipients. They do this *via* a process of paired or pooled donation (11). This means that often people who want to donate a kidney to a relative or friend but are not a (good) match will instead donate their kidney to what is called the “kidney donor pool”. This system enables a much more comprehensively assessed matching process in terms of blood group or tissue type rather than just on relationship to the recipient (1–4).

Live kidney donation continues to be promoted as a better option for patients with kidney failure and is associated with better outcomes (more effective matching profiles mean kidneys function better, last longer, with less risk of rejection) (12, 13) and is cost effective (patients normally receive a transplant quicker, cost the health service less, and if well-planned, patients can often avoid costly dialysis) (14, 15).

For example, in 2022 kidney transplantation resulted in a cost-benefit of about £27,155.8 per annum compared to dialysis, thus accruing benefits to both patients and national health services (1–4). Also, Gibbons et al.'s (16) analysis of 12 months' post-transplant cross-sectional survey data suggested a better quality of life and treatment satisfaction for patients who received a kidney transplant from live donors compared to those who received deceased donor organs. Furthermore, the risks to live donors are minimal – data suggest that mortality is on par with routine surgery, which equates to about 1/3,000 for kidney donors, 1/200 for right liver lobe donors and 1/5,000 for left lateral liver donors (17–20).

In spite of such developments there remains a critical shortage of available organs for transplantation to meet the health needs of over 7,000 people on the transplant waiting lists in the UK; with about three people estimated to die every day while awaiting an organ transplant (21). There is also emerging evidence that although the number of live donors has increased over the past 20 years, more recently these numbers plateaued (around 1,000 donors per year, accounting for around 35% of overall transplant activity in 2019) (22, 23).

In addition, the world continues to be burdened with end-stage kidney disease due to increasing population size and age, as well as increasing prevalence of associated co-morbid chronic health conditions such as diabetes, cardiovascular disease and hypertension (24). Variation in health systems in addition to public awareness, and cultural and ethnic differences in support for donation, mean that uptake of live donation can vary dramatically in and between countries (25). Although increasing the number of live donations remains a global health priority, interventions designed to increase live donation are poorly understood, lack an evidence base, and do not easily translate across diverse populations, so the unmet health needs and the economic burden of those awaiting transplant remain high (26, 27).

While research continues at pace to expand the numbers of deceased organs available for transplant including organ preservation (28), public attitudes (29), family behaviors (30, 31), professional training (32), law and policy changes (21, 33) and awareness and understanding in and between minority and faith perspectives, (21, 26, 31, 34, 35); investigations into (changing) attitudes and motivations to become a living donor have been much more limited. In 2018, NHSBT also published a warning after living donation hit an 8-year low (36). Studies which have investigated public perspectives on living donation have identified preconceived ideas, misconceptions, concerns about the risks involved, lack of trust in systems, cultural beliefs and personal values as potential barriers to live organ donation (37–39). However, these studies were conducted some time ago, are likely not reflective of what is achievable today in living donation, and did not aim at characterize who is more likely to want to become a living donor and why. The aim of this study was to better understand the factors that influence intentions to become a living kidney donor to inform current and future policy interventions designed to increase the number of live donors.

## 2. Article materials and methods

### 2.1. Questionnaire and data

This study was undertaken as part of a wider national evaluation into the evolving organ donation system in England following the introduction of a soft opt-out policy in May 2020 (40). Following ethics approval for the study from the LSHTM ethics committee (Ref: 26427) and HRA (Ref: 21/NW/0151), NHSBT's national organ donation survey data were shared with the research team which included a series of questions related to live donation. The key question asked and the response options are shown in Box 1. This question was the focus of the current analysis.

Box 1 **Key question of analysis.**

In which, if any, of the following circumstances would you consider donating one of your kidneys while you were alive? Please select all that apply.

**Options:**

- I would consider becoming a living kidney donor for a family member
- I would consider becoming a living kidney donor for a friend
- I would consider becoming a living kidney donor for someone I don't know
- I am unlikely to consider becoming a living kidney donor.
- I would never become a living kidney donor
- Not applicable - I have been a living kidney donor/recipient
- Don't know

The data comprised of eleven repeated cross-sectional surveys undertaken from August 2015 to November 2021 (*n* = 19,011) with an average of eight months' interval in between surveys. The same questions were administered to a new sample of respondents at each of the serial surveys. The data were collected as part of the organ donation attitudinal tracker survey commissioned by NHSBT.

The participants were recruited from the online panel of the survey organization called Kantar. The online panel consists of recruited adults aged 16 years and over who have given their explicit permission to be contacted about surveys. The panelists were recruited using telephone recruitment from small area census statistics and Postcode Address File (PAF) in England. These areas are of similar population sizes formed by the combination of wards with the constraint that each point must be contained within a single Government Office Region. The total size of the panel is about 30,000. The survey participants were invited to answer the survey using a quota sampling of individuals with random locational sample selection. Each quota was set based on national census data on age, education and geographical region. Different quota was set for each round of survey so there were not duplicate responses by the same individual in the serial surveys. Panelists were invited by email to answer the survey. They were offered small financial rewards after completing the surveys. The samples were weighted to represent the adult population of England who are 16 years of age and older.

We excluded all responses in the first three rounds of survey because the key question of focus (see Box 2.1) was not asked during these surveys (*n* = 4,110). All respondents who resided in Wales (*n* = 200) during the survey, and all those who did not provide information on their age were excluded from the dataset (*n* = 194). In addition, respondents who had been a living kidney donor or recipient were excluded because their responses were not related to future intentions (*n* = 229). A total sample of 14, 278 was used for the analysis.

### 2.2. Statistical analysis

Statistical analysis was done using R (41). As well as undertaking an overall analysis using all those who would consider donating a kidney to a family member, a friend or an unknown person, and those not willing; sub-analysis was done focusing on those who would consider donating to a family member and those not willing; those who will consider donating to a friend and those who are unwilling; as well as those who will consider donating to an unknown person and those who would be unwilling. Frequency distributions, weighted percentages, means and standard deviations were used to describe the characteristics of respondents. The relationships between the demographic characteristics (age, sex, ethnic origin, number of children in household, religion, occupation, awareness of organ donation publicity, and support for organ donation) of respondents and their intentions to become a living kidney donor were determined using Pearson's *x*^2^ test.

We used random forest model, a machine learning approach, to identify important factors influencing intentions, and predicting decisions to become a living donor. Applications of the random forest model in the fields of economics (42), and health and environmental sciences (43) have increased rapidly in recent years. Studies that have compared results of random forest model to other approaches either found similar results (44) or that the random forest model algorithm perform well in predicting decisions compared to approaches such as ordinary least squares regression (45) and logistic regression (46, 47). This is because of its adaptability to both linear and non-linear distributions, allowing complex interactions between characteristics and because it requires no prior model specification. We use random forest model because in addition to prediction accuracy, the random forest model enables identification of subgroups and their decision formation patterns (decision tree), a feature that cannot be obtained from one traditional methodology.

The random forest model is an ensemble of decision-trees which involves recursively partitioning a given data into two groups based on the response distribution until a predetermined stopping condition is achieved (48). The forest repeats this process many times using random subsets of the observations and variables. Hence, random forests are less prone to overfitting than a single decision tree (44). Based on how the partitioning and stopping criteria are set, the model can be designed for both categorical outcome variables and continuous outcome variable of interest. For a categorical outcome problem, as in the current study, a commonly used splitting criterion is entropy (49). At a given internal node of the decision tree, entropy is given as:


(2.1) $$\begin{array}{l} E=-\overset{c}{\underset{i=1}{\sum }}p_{i}x\log\left(p_{i}\right) \end{array}$$

Where c is the number of unique classes or splits and *p*_*i*_ is the probability of each given class or split. The value of the probability is maximized in order to gain the most information at every split of the decision tree.

Based on available data, literature and intuition, the variables included in the model, their definitions and measurements are shown in Appendix 1. Individuals were grouped according to the quintile of their predictions, and the mean characteristics were presented by quintile to allow a better understanding of the relationship between the variables and the predicted intentions to become a living donor.

## 3. Results

### 3.1. Characteristics of respondents

Of a total sample of 14, 278 included in the analysis, 58.8% (*n* = 8,400) would consider becoming a living kidney donor while the remaining 41.2% (*n* = 5,878) would not consider becoming a living kidney donor. The characteristics of respondents (age, gender, ethnic origin, number of children in household and occupation) are shown in Table 1. Apart from the number of children in respondents' household, and ethnic origin, the differences in the aforementioned characteristics were statistically significant at 5% level across the categories. For instance, the average age of all respondents was 43 years (standard deviation 17). The average age was a year less for those who would consider donating their kidney than for those not willing to become a living donor. The difference in age was statistically significant at the 1% level. About 51% (*n* = 7,528) of the respondents were female. The proportion of females who would consider becoming a living kidney donor was about 10% higher compared to males. The differences were statistically significant at 1% level. The majority, about 92.8% (*n* = 11,736) of respondents self-described as being ethnically White. The level of awareness of organ donation publicity was modest at 37.1% (*n* = 5,520). The level of awareness of organ donation publicity campaigns for those who would consider becoming a living donor was 40.5% (*n* = 3,561), this is 8% higher compared to those who would not consider becoming a living kidney donor. Support for organ donation was high among the respondents with 78.1% (*n* = 10,966) in overall support. Support for organ donation was 20% higher for those respondents who would consider donating their kidney to either a family member, a friend or an unknown person (86%) compared to those who were not willing to become a living kidney donor.

**Table 1:** Characteristics of respondents.

**Characteristics**	**Would not consider becoming a living donor [*n* = 5,878 (41.2%)]**	**Would consider becoming a living donor [*n* = 8,400 (58.8%)]**	**Total** **(*n* = 14,278)**	***P*****-value** **(χ^2^ test)**
Age, mean (SD%)	44 (17%)	43 (17%)	43 (17%)	0.000
**Sex**, ***n*** **(%)**				
Male	3055 (53.1%)	3683 (45.57%)	6738 (48.68%)	0.000
Female	2821 (46.9%)	4707 (54.29%)	7528 (51.22%)	
Prefer not to say	1 (0.03%)	0 (0%)	1 (0.01%)	
Other	1 (0.02%)	10 (0.14%)	11 (0.09%)	
**Ethnic origin**, ***n*** **(%)**				
Other	1085 (7.9%)	1457 (6.77%)	2542 (7.24%)	0.087
White	4793 (92.1%)	6943 (93.23%)	11736 (92.76%)	
**Number of children in household**, ***n*** **(%)**				
1	739 (10.98%)	1259 (14.03%)	1998 (12.76%)	0.242
2	602 (8.9%)	1034 (11.53%)	1636 (10.44%)	
3	170 (2.07%)	302 (3.13%)	472 (2.69%)	
4	55 (0.63%)	77 (0.84%)	132 (0.75%)	
5	19 (0.32%)	15 (0.17%)	34 (0.23%)	
More than 5	10 (0.08%)	15 (0.16%)	25 (0.13%)	
No response	4283 (77.01%)	5698 (70.15%)	9981 (73%)	
**Religion**, ***n*** **(%)**				
Christianity	2575 (47.17%)	3974 (50.43%)	6549 (49.07%)	0.000
Islam	475 (4.06%)	488 (2.79%)	963 (3.31%)	
Hinduism	154 (1.25%)	239 (1.35%)	393 (1.31%)	
Sikhism	54 (0.47%)	103 (0.57%)	157 (0.53%)	
Buddhism	41 (0.74%)	58 (0.73%)	99 (0.73%)	
Judaism	35 (0.7%)	53 (0.72%)	88 (0.71%)	
Other	115 (1.84%)	165 (1.99%)	280 (1.93%)	
No response	2429 (43.77%)	3320 (41.43%)	5749 (42.4%)	
**Occupation**, ***n*** **(%)**				
High managerial, administrative or professional	375 (5.25%)	688 (7.2%)	1063 (6.39%)	0.000
Intermediate managerial, administrative	1273 (20.66%)	2036 (22.79%)	3309 (21.91%)	
Supervisor, clerical, junior managerial	1515 (24.77%)	2156 (25.27%)	3671 (25.06%)	
Skilled manual worker – e.g., mechanic,	1072 (20.75%)	1576 (21.19%)	2648 (21.01%)	
Semi-skilled or unskilled manual worker	820 (14.9%)	1091 (13.82%)	1911 (14.27%)	
Housewife/househusband	160 (2.68%)	191 (2.48%)	351 (2.57%)	
Unemployed	453 (8.74%)	402 (5.13%)	855 (6.63%)	
Student	205 (2.22%)	246 (2.01%)	451 (2.1%)	
Do not wish to answer	3 (0.01%)	5 (0.04%)	8 (0.03%)	
No response	2 (0.01%)	9 (0.06%)	11 (0.04%)	
**Awareness of organ donation publicity**, ***n*** **(%)**				
Yes	1959 (32.25%)	3561 (40.46%)	5520 (37.05%)	0.000
No	3575 (62.31%)	4503 (55.48%)	8078 (58.31%)	
Don't know	344 (5.44%)	336 (4.06%)	680 (4.63%)	
**Support for organ donation**, ***n*** **(%)**				
Support organ donation	3830 (67.06%)	7136 (86.11%)	10966 (78.19%)	0.000
Indifferent	1523 (24.93%)	897 (9.9%)	2420 (16.15%)	
Oppose organ donation	303 (4.63%)	297 (3.23%)	600 (3.81%)	
No response	222 (3.38%)	70 (0.76%)	292 (1.85%)	

% represents weighted percentage; SD represent standard deviation.

**Figure 1:**
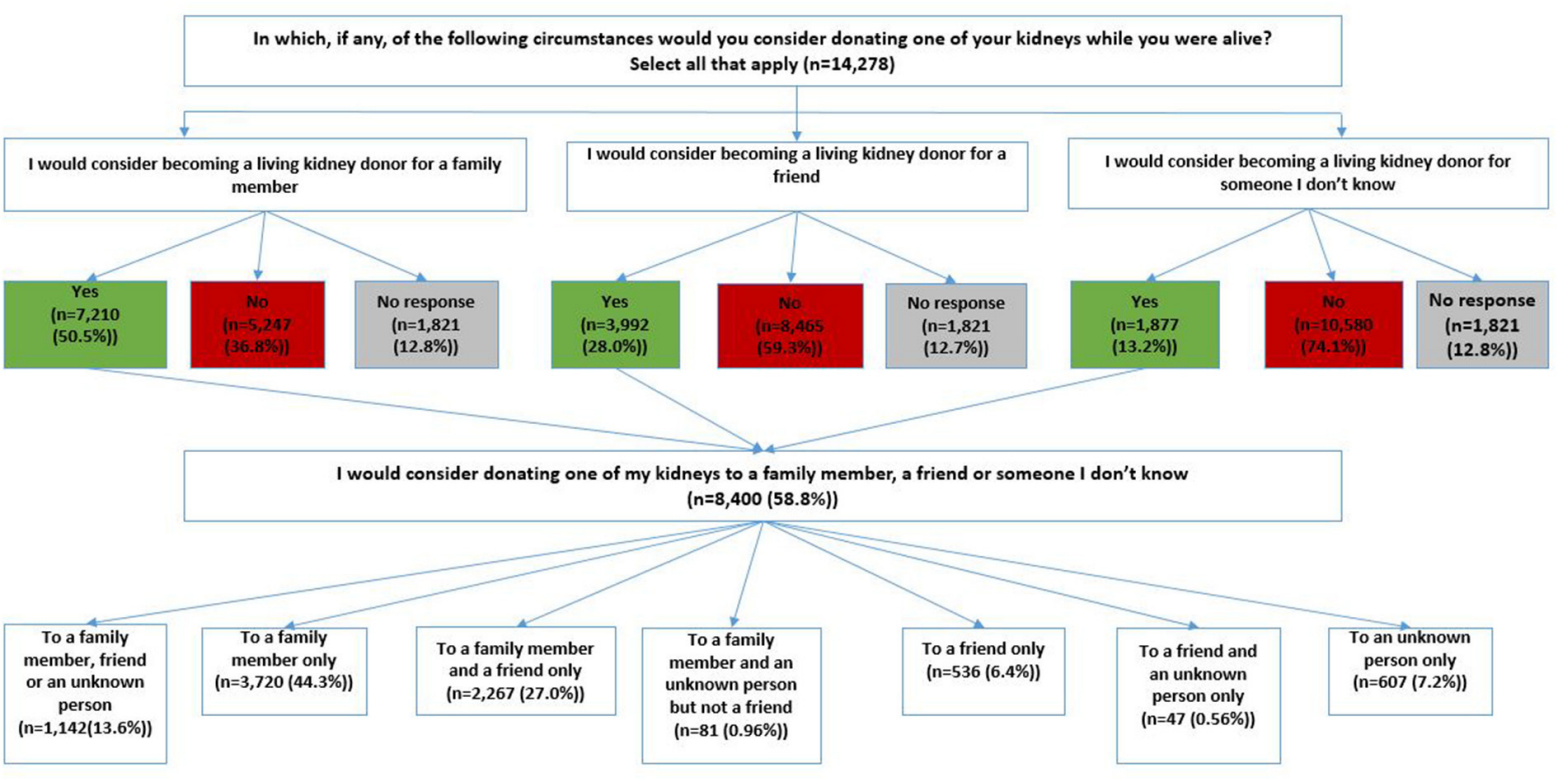
Sample distribution of respondents.

Of the 58.8% (*n* = 8,400) who would consider becoming a living donor, 50.5% (*n* = 7,210) would consider becoming a living kidney donor for a family member, 28% (*n* = 3,992) would consider donating their kidney to a friend, and 13.2% (*n* = 1,877) would consider donating to an unknown person (Figure 1). Also, 44.3% (*n* = 3,720) would only consider donating to a family member; 6.4%(n=536) would only consider donating to a friend, and 7.2% (*n* = 607) would only consider donating to an unknown person (Figure 1).

Table 2 show the characteristics of respondents who would consider donating to a family member, a friend or an unknown person. The results show that those who would consider donating a kidney to a family member were 3 years older, with an average age of 47 years, compared to those who would consider donating to a friend and an unknown person. Male respondents were more likely to consider donating a kidney to a friend (48%, *n* = 1,799) or an unknown person (48%, *n* = 869); while females were more likely to consider donating to a family member (57%, *n* = 4,215). These differences were statistically significant at 1% level. Overall, the majority of respondents who self-described as being ethnically White were more likely to donate to a family member (94%, *n* = 6,119), and to a friend (94%, *n* = 3,387), compared to an unknown person (92%, *n* = 1,523). The level of awareness of organ donation publicity was comparatively higher among respondents who would consider donating to a friend, 40%, (*n* = 1,699). Support for organ donation were generally high for all living donor intended categories, about 88% (Table 2).

**Table 2:** Characteristics of respondents who would consider donating to a family member, a friend or an unknown person.

**Characteristics**	**I would consider donating my kidney to**			***P*** **-value (*X*^2^ test)**
	**Family [*****n*** **= 7,210 (50.5%)]**	**Friend [*****n*** **= 3,992(28.3%)]**	**Unknown [*****n*** **= 1,877(13.2%)]**	
Age, mean (SD)	47.38 (17.51)	43.59 (17.71)	44.39302 (17.83)	0.000
**Age groups, n (%)**				
16–29	1821 (18.71%)	1314 (25.73%)	595 (24.82%)	
30–49	2433 (33.45%)	1351 (34.93%)	647 (34.6%)	0.000
50–64	1908 (24.68%)	896 (21.89%)	423 (22.22%)	
65 and over	1048 (23.16%)	431 (17.45%)	212 (18.36%)	
**Sex**, ***n*** **(%)**				
Male	2989 (43.15%)	1799 (47.54%)	869 (48.45%)	0.000
Female	4215 (56.74%)	2186 (52.26%)	1000 (50.99%)	
Other	6 (0.11%)	7 (0.20%)	8 (0.56%)	
**Ethnic origin**				
Other	1091 (5.79%)	605 (5.97%)	354 (7.55%)	0.000
White	6119 (94.21%)	3387 (94.03%)	1523 (92.45%)	
**Number of children in household**, ***n*** **(%)**				
1	978 (12.81%)	578 (14.11%)	305 (15.3%)	
2	825 (10.78%)	434 (10.33%)	226 (11.46%)	
3	240 (2.89%)	152 (3.45%)	78 (3.99%)	0.198
4	56 (0.65%)	35 (0.83%)	16 (0.84%)	
5	9 (0.11%)	9 (0.21%)	1 (0.01%)	
More than 5	12 (0.15%)	4 (0.09%)	4 (0.22%)	
No response	5090 (72.61%)	2780 (70.98%)	1247 (68.18%)	
**Religion**, ***n*** **(%)**				
Christianity	3454 (50.98%)	1769 (49.45%)	868 (49.45%)	
Islam	338 (2.05%)	208 (3.28%)	114 (3.28%)	
Hinduism	176 (1.11%)	96 (1.86%)	67 (1.86%)	0.000
Sikhism	74 (0.5%)	48 (0.73%)	31 (0.73%)	
Buddhism	40 (0.56%)	31 (0.79%)	14 (0.79%)	
Judaism	47 (0.75%)	23 (0.58%)	12 (0.58%)	
Other	138 (1.95%)	96 (1.94%)	34 (1.94%)	
No response	2943 (42.1%)	1721 (41.36%)	737 (41.36%)	
**Occupation**, ***n*** **(%)**				
High managerial, administrative or prof	551 (6.61%)	325 (7.24%)	159 (7.43%)	
Intermediate managerial, administrative	1717 (22.52%)	990 (22.93%)	434 (21.08%)	
Supervisor, clerical, junior managerial	1909 (25.91%)	1036 (25.45%)	434 (22.35%)	0.000
Skilled manual worker - e.g., mechanic,	1367 (21.36%)	729 (20.51%)	357 (21.83%)	
Semi-skilled or unskilled manual worker	951 (14.06%)	481 (13.4%)	246 (15.22%)	
Housewife/househusband	174 (2.61%)	85 (2.37%)	44 (2.48%)	
Unemployed	345 (5.11%)	193 (5.32%)	112 (6.47%)	
Student	182 (1.69%)	142 (2.60%)	86 (2.98%)	
Do not wish to answer	5 (0.04%)	3 (0.05%)	2 (0.09%)	
No response	9 (0.07%)	8 (0.12%)	3 (0.09%)	
**Awareness of publicity**, ***n*** **(%)**				
Yes	2889 (38.15%)	1699 (39.99%)	860 (44.37%)	
No	4016 (57.55%)	2107 (55.11%)	943 (51.82%)	0.000
Don't know	305 (4.30%)	186 (4.90%)	74 (3.80%)	
**Support for organ donation**, ***n*** **(%)**				
Support organ donation	6258 (87.84%)	3559 (89.87%)	1650 (88.34%)	
Indifferent	720 (9.25%)	299 (7.05%)	153 (7.68%)	0.000
Oppose organ donation	177 (2.18%)	112 (2.62%)	62 (3.49%)	
No response	55 (0.73%)	22 (0.46%)	12 (0.48%)	

### 3.2. Factors influencing intentions to become a living donor

**Figure 2:**
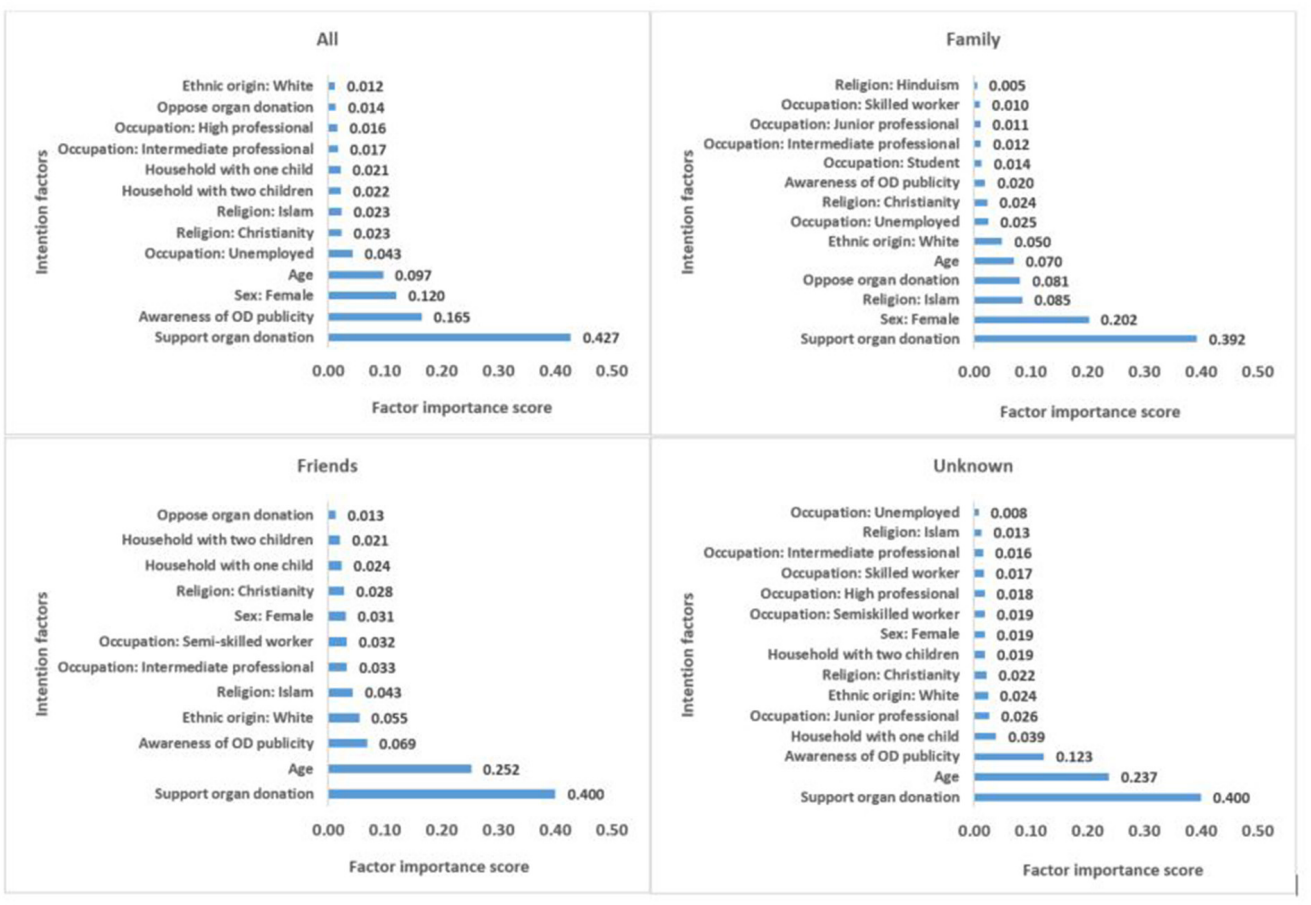
Important factors influencing intentions to become a living donor by recipient type.

The most important factors influencing intentions to become a living donor are shown in Figure 2. The results are presented separately for the total sample, those who would consider donating to a family member, a friend and an unknown person. The vertical axis shows the factor importance score–the figures represent the number of times a given factor/variable is used by the random forest to inform predicted intention to become a living donor. In the modeling process, the importance score represents the number of times a given variable is used to split on in the trees in the forest. The sum of all the importance scores is equal to 1 (100%). Out of the 25 factors/variables included in the model, the algorithm identified 13 important intention factors for the total sample, 14 important intention factors for the sub-sample who would consider donating their kidney to a family member, 12 important intention factors for those who would consider donating to a friend and 15 important factors for those who would consider donating to an unknown person (Figure 2).

The results from the total sample show that the most important factor that informs living donor intentions is support for organ donation. This is followed by awareness of organ donation publicity. These factors precede other important sociodemographic factors such as gender, age, occupational status, religion, number of children in household and ethnic origin, in reducing order of importance. A similar trend of factor importance was found in the subgroup analysis, however, the order of importance and the factor scores differed somewhat across the subcategories. For all the subgroups, the most important living donor intention factor is support for organ donation. While this was followed by gender in the case of those who intend to donate to a family member; age was the second most important factor taken into consideration by those who intend to donate to a friend or an unknown person (Figure 2).

**Figure 3:**
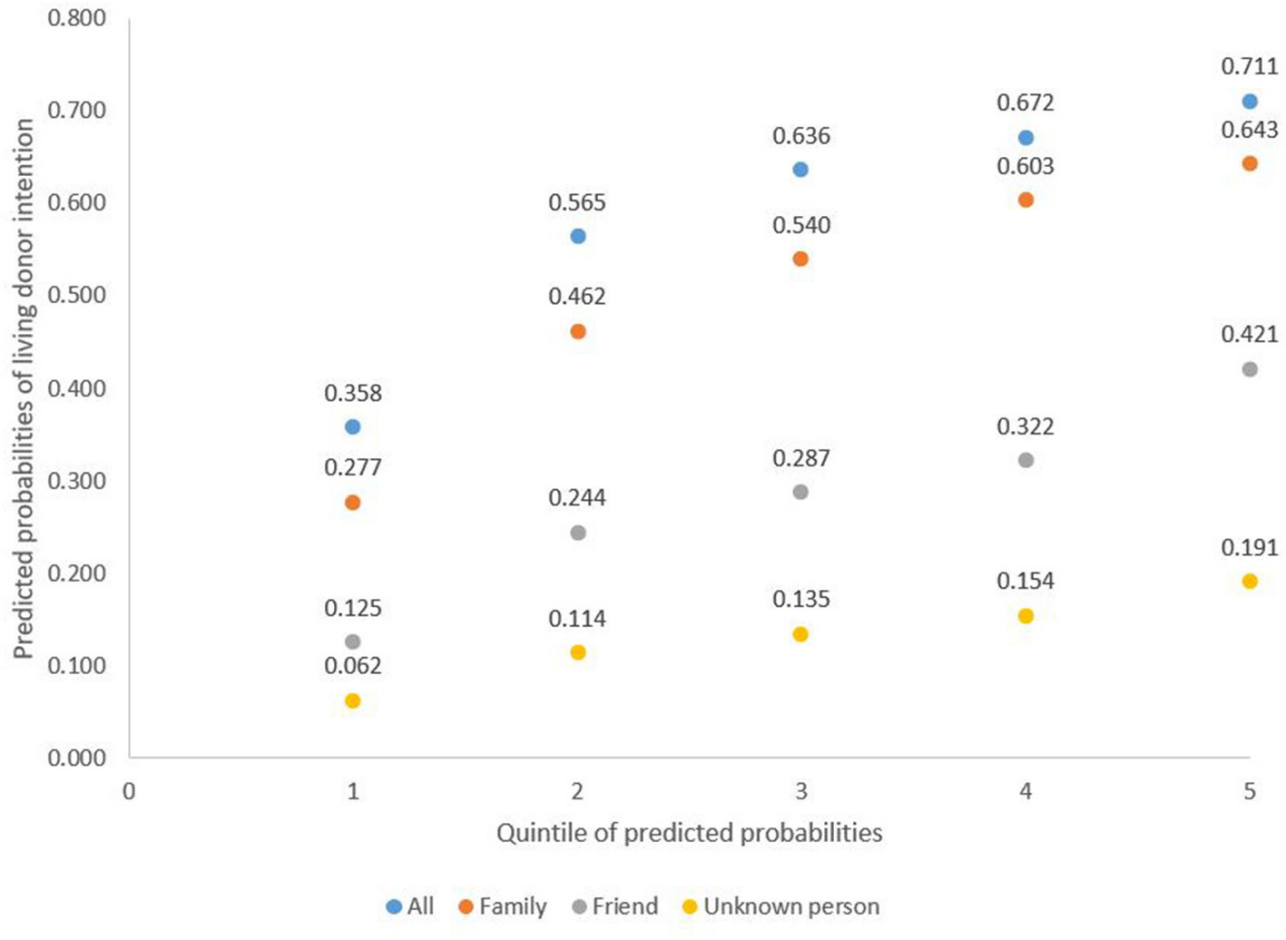
Mean prediction of likelihood of intention to become a living donor per quintile of predicted intention probabilities.

The mean predicted propensity for living donation by quintile for each of the estimated models are shown in Figure 3. The results show that the mean predicted propensity for living donation in the first quintile is 35.8% (in the total sample), compared to 71.1% for those in quintile 5 (Figure 3).

Results for the propensity to donate to anyone (total sample) follows a similar pattern to the propensity to donate to a family member. There is considerable heterogeneity with the predicted propensity for living donation, which increased substantially from quintile 1 to quintile 5. The propensity to donate to a friend or unknown person is lower and displays less heterogeneity, increasing modestly from quintile 1 to quintile 5 (Figure 3).

Table 3 shows the mean estimates of covariates/factors by quintile of predicted propensity to living donation. The results show that females are positively associated with predicted propensity for living donation.

**Table 3:** Mean/proportional estimate of covariates by quintile of predicted probability of living donation (Total sample).

	**Quintile1**		**Quintile2**		**Quintile3**		**Quintile4**		**Quintile5**	
**Covariates**	**Mean/** **Proportion**	**Standard error**	**Mean/** **proportion**	**Standard error**	**Mean/** **proportion**	**Standard error**	**Mean/** **proportion**	**Standard error**	**Mean/** **proportion**	**Standard error**
Sex: Female	0.49	0.008	0.16	0.008	0.40	0.008	0.76	0.008	0.83	0.008
Age	43	0.304	50	0.304	48	0.304	42	0.304	33	0.304
Household with one child	0.11	0.006	0.09	0.006	0.11	0.006	0.16	0.006	0.23	0.006
Household with two children	0.08	0.006	0.09	0.006	0.10	0.006	0.14	0.006	0.16	0.006
Household with three children	0.04	0.003	0.02	0.003	0.03	0.003	0.05	0.003	0.02	0.003
Household with four children	0.01	0.002	0.01	0.002	0.01	0.002	0.01	0.002	0.01	0.002
Household with five children	0.01	0.001	0.00	0.001	0.00	0.001	0.00	0.001	0.00	0.001
Ethnic origin: White	0.74	0.007	0.88	0.007	0.85	0.007	0.84	0.007	0.81	0.007
Religion: Christianity	0.42	0.009	0.38	0.009	0.56	0.009	0.50	0.009	0.43	0.009
Religion: Islam	0.13	0.005	0.06	0.005	0.06	0.005	0.04	0.005	0.04	0.005
Religion: Hinduism	0.02	0.003	0.01	0.003	0.03	0.003	0.04	0.003	0.04	0.003
Religion: Sikhism	0.02	0.002	0.01	0.002	0.01	0.002	0.01	0.002	0.01	0.002
Religion: Buddhism	0.01	0.002	0.01	0.002	0.01	0.002	0.01	0.002	0.01	0.002
Religion: Judaism	0.01	0.001	0.01	0.001	0.01	0.001	0.01	0.001	0.00	0.001
Occupation: High professional	0.04	0.005	0.05	0.005	0.08	0.005	0.08	0.005	0.12	0.005
Occupation: Intermediate professional	0.16	0.008	0.26	0.008	0.26	0.008	0.18	0.008	0.31	0.008
Occupation: Junior professional	0.25	0.008	0.24	0.008	0.26	0.008	0.31	0.008	0.22	0.008
Occupation: Skilled worker	0.20	0.007	0.18	0.007	0.19	0.007	0.20	0.007	0.16	0.007
Occupation: Semiskilled worker	0.17	0.006	0.12	0.006	0.12	0.006	0.15	0.006	0.11	0.006
Occupation: Housewife husband	0.03	0.003	0.02	0.003	0.02	0.003	0.03	0.003	0.02	0.003
Occupation: Unemployed	0.11	0.004	0.11	0.004	0.06	0.004	0.02	0.004	0.01	0.004
Occupation: Student	0.04	0.003	0.01	0.003	0.03	0.003	0.03	0.003	0.05	0.003
Awareness of organ donation publicity	0.19	0.009	0.27	0.009	0.46	0.009	0.41	0.009	0.62	0.009
Support organ donation	0.01	0.003	0.86	0.003	0.99	0.003	1.00	0.003	0.99	0.003
Oppose organ donation	0.12	0.004	0.06	0.004	0.02	0.004	0.01	0.004	0.01	0.004

The proportion of females in the first quintile was 49%, this reduced to 16% in the second quintile but increased thereafter to 83% in quintile 5. Age was negatively related with predicted propensity for living donation with a mean of 43 years in the first quintile, which increased to 50 years in the second quintile. The mean age then decreased continuously, reaching 33 years in quintile 5 (Table 3). Support for organ donation is positively related to predicted propensity for living donation. The proportion which had support for organ donation was 1% in the first quintile and raised to 99% in the fifth quintile. On the contrary, opposition to organ donation was negatively associated with predicted propensity for living donation. The proportion of those who oppose organ donation in the first quintile was 12% and reduced to 1% in the fifth quintile. Awareness of organ donation publicity was positively associated with predicted propensity for living donation, with 19% level of awareness in the first quintile which rises to 62% level of awareness in quintile 5 (Table 3). The results for family, friend and unknown person samples can be found in Appendix 2–4.

**Figure 4:**
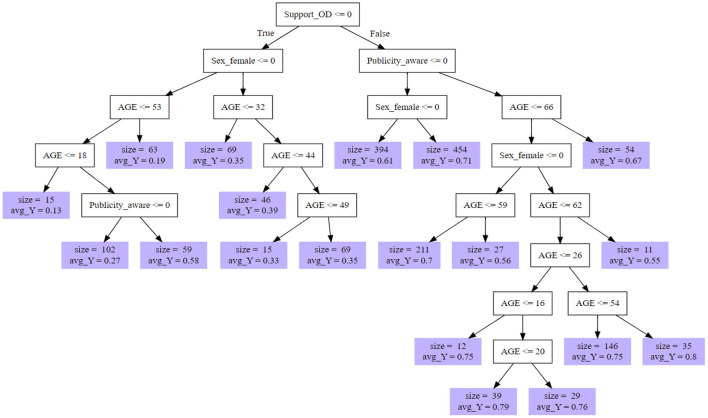
Pruned decision tree for predicted propensity for living kidney donation based on top four most important factors.

The random forest decision tree distribution showed a complex decision formation process that are highly individualized in nature, based on the identified factors, in informing intention to become a living kidney donor. Although we could not show all the decision trees in the forest, Figure 4 shows pruned decision trees based on the first four most important factors - that is, support for organ donation, awareness of organ donation publicity, gender and age.

The decision nodes show the number and size of subgroups as well as their predicted propensity for living donation (see violet nodes). For instance, the first four most important factors result in 19 subgroups with similar propensities for living donation. The results also show that although some people may oppose organ donation, they might consider becoming a living donor as indicated in the left hand nodes of the decision tree (Figure 4).

## 4. Discussion

We identified important factors that influence intentions to become a living kidney donor. They include support for organ donation, awareness of organ donation publicity, gender, age, occupation, religion, number of children in the household, and ethnic origin, in reducing order of importance. Support for organ donation, awareness of public campaigns, being younger, female and unemployed were all positively associated with people who reported being happy to become a living kidney donor. Our analysis also highlighted the complexity and individual nature of people's intentions to become a live kidney donor. Decision-making was highly personal and dependent on a range of factors, and likely a result of people's experiences as well as personal preferences and characteristics. For example, we noted a small number of cases where individuals do not support organ donation but nonetheless would be comfortable to donate a kidney to a relative, friend or even an unknown person. This may indicate that intentions to become a living donor are sometimes a result of individual circumstances including life events, not solely determined by demographics, and also that a person's intentions to become a live donor may change over time.

In a global context, interventions designed to increase living organ donation have varied considerably, largely due to factors such as health system capacity, health of the population, policy contexts, trust in government, and an established organ donation (including research) culture (50–53).

In the UK and countries with similar healthcare systems research has more recently focused on live donor trends in relation to deceased organ donation (54). Some evidence suggests that as the number of deceased donations has increased, the number of live donations has fallen (55). We do not yet fully understand why this is happening, but a recent multi-stakeholder call to action has highlighted the need to optimize live donation as a priority, while at the same time listing some key factors, in particular, the need to demystify the risks of live donation, and develop better education for potential donors and recipients (56).

Increasing the number of live donations is seen by health authorities as vital to help address the substantial inequalities apparent in organ donation and transplant. In the UK people from Black, Asian and minority ethnic backgrounds are overrepresented on the transplant waiting lists, overrepresented on the opt-out organ donor register, and are more likely to say no to deceased organ donation (57). Improving the uptake of live donation across these populations is widely agreed will do more to help level-up inequalities across these populations than any other single intervention (1–4).

Previous study findings have highlighted the important role played by ethnicity and religion in decisions associated with deceased organ donation (26, 35). Our findings show that ethnic origin and religion are of less importance in the case of living kidney donation. Although the proportion of ethnic minorities in the surveys was small (7.4%) and the surveys were not specifically designed to look at their perspectives, our findings are consistent with Siegel et al. (39) who employed planned behavior and a vested interest approach to explore the differences in intentions to become a living and deceased organ donor. They concluded that intention to become a living and deceased organ donor are very different and require independent examination and further study. For example, a clear difference in practice is that the living donor gives their own consent to the surgery which, unlike in the case of deceased organ donation, cannot be overridden by relatives.

Finally, our findings indicate that people are perhaps unsurprisingly more likely to want to donate to a relative only. This may indicate a lack of awareness and understanding of the process of live kidney donation since it is often the case that people do not donate directly to their relative but to a donor pool, and that importantly this process actually enables better matching and outcomes for recipients (39, 58).

### 4.1. Strengths and limitations

This study applied random forest model, a machine learning approach, to identify and predict the factors that influence intentions of becoming a living kidney donor to help inform present and future health communication programmes and interventions aimed at increasing living organ donation. The random forest approach was used because it does not require prior correct model specification, prevents overfitting of the model and produces accurate estimates of measurement errors. For some of the analysis we split the dataset into three categories based on whether respondents' intent to donate their kidney to a family member, a friend or an unknown person. Grouping the dataset this way provided a more detailed understanding of the data routinely collected by NHSBT and helped develop understanding of the factors that can inform people's intentions to become a living kidney donor either to a family member, friend or unknown person. This is important as the UK is currently a world leader in paired/pooled living kidney donation through the UK Living Kidney Sharing Scheme (UKLKSS) which enables family members to donate to a “donor pool” rather than directly to their relative (1–4). The living donor is unlikely to ever know who received their kidney but will be reassured that their relative is better matched *via* blood and tissue type and will wait less time for a kidney.

The main limitation of this study is that the authors were not involved in the questionnaire design or data collection and so were limited in their analysis to a small number of questions asked about living donation as part of a series of cross-sectional national surveys looking generally at attitudes to organ donation. This limited the number of variables that could be included in the model. Overall, our model predicted 71.1% of the factors that informs intentions to become a living kidney donor. Future studies should help to account for the remaining 28.9% of the factors not accounted for in this study. Also, the sampling might not be sufficient to capture thorough population level distributions and may involve biases. The surveys were not longitudinal and so we were unable to look at changes over time including patterns or events which may have influenced public attitudes to live donation, e.g., changes in organ donation policy – for example, those introduced in England in May 2020.

### 4.2. Recommendations and future research

The study results demonstrate the need to promote health communication campaigns to increase public awareness of living organ donation as well as educating the public on existing structures and processes involved in becoming a living donor. Such interventions could target adult population who are below the age of 45 years. There remain large gaps in knowledge in relation to motivations and eventual behavior related to live donation, for example ethnic minority perspectives, the personal views and experiences of those who have become living donors, those who have requested a live donation from a relative or friend, and importantly more detailed data on why people say they do not want to become live donors, or donate to certain people, for example, those with serious drug use, convicted of serious crime or those who are perceived to have “abused” a previous organ following transplantation; why people refuse the offer of a live donation, and how perspectives and attitudes may change over time. Plus, we have very little evidence about the ethical or positive and negative psychological impacts or consequences of living donation. For example, what are the experiences of donating to a relative if the relationship breaks down or they do not look after the kidney as well as the donor would expect?

The survey could be improved by including additional questions such as educational level of respondents, motivations/demotivations to becoming a living kidney donor and their experiences with living donation, among others. Also, the survey could be implemented as panel survey instead of repeated cross-sectional survey with different sample of respondents for each survey wave. That would help to measure changes in behavior and intentions to become a living donor over time. The online survey could be complemented with paper-based survey *via* post to targeted respondents within the selected same small area census statistics and Postcode Address File (PAF) in England to reduce possible sample selection bias. Although the results show that ethnic origin is of less importance in the case of living kidney donation, future surveys could be designed to purposively increase response from ethnic minority groups in order to fully capture their perspectives. Future research needs to take a more complex system perspective including looking at what can be done to increase the donor pool and make more live donor organs available for transplant, complimented with longitudinal data investigating patients' outcomes and cost effectiveness.

## 5. Conclusion

Live kidney donation remains the best treatment for end-stage renal diseases as it is cost-effective, and a preferred choice for many patients compared with other forms of treatment such as dialysis. Nonetheless, despite investments, the number of people becoming live kidney donors has plateaued in recent years. Our analysis has identified some of the key factors which are likely to influence people to be potentially willing to become a living kidney donor and at the same time (re)established the complexity of decision making around this highly personal and sometimes controversial topic. There are gaps in public knowledge and awareness of live donation in general, and how it is likely to come about in practice. Addressing some of these gaps may facilitate greater uptake of live organ donation. Nonetheless, additional research is required in order to better understand motivations toward live donation and ensure those who are eligible and want to become live organ donors are able to do so in future.

## Data availability statement

The data analyzed in this study are subject to licenses/restrictions. The authors do not have the permission to publish the dataset. Requests to access these datasets should be directed to https://www.nhsbt.nhs.uk.

## Ethics statement

The studies involving human participants were reviewed and approved by LSHTM Ethics Committee (Ref: 26427) and HRA (Ref: 21/NW/0151). The participants provided their informed consent to participate in this study.

## Author contributions

PB conceptualized the analysis, performed data analysis, drafted the first version of the manuscript, and finalized the manuscript. LM, MA-H, and JB conceptualized the analysis, supported drafting the manuscript, and provided feedback on early versions. SO'N conceptualized the analysis, supported data analysis, provided feedback on early versions, and approved the final version of the manuscript. JN and NM conceptualized the analysis, provided feedback on early versions, and approved the final version of the manuscript. All authors contributed to the article and approved the submitted version.
