# Supplementary material for: A machine-learning approach to estimating public intentions to become a living kidney donor in England: Evidence from repeated cross-sectional survey data
Source: Front Public Health. 2023 Jan 4;10:1052338. doi: 10.3389/fpubh.2022.1052338 (PMC9846224; doi:10.3389/fpubh.2022.1052338)
Supplement: Supplementary file 1 [file Data_Sheet_1.docx]

Supplementary Material

# Supplementary Tables

Appendix 1: Variables, definitions and their measurement

| **Variable** | **Definition and measurement** |
| --- | --- |
| ***Depended variable*** |  |
| Intentions | A dummy variable measured at four level:   1. 1 if the individual respondent answered yes to consider becoming a living kidney donor for a family member, and 0 otherwise; 2. 1 if the individual respondent answered yes to consider becoming a living kidney donor for a friend; 3. 1 if the individual respondent answered yes to consider becoming a living kidney donor for someone they don’t know; and a pooled indicator 4. 1 if the individual respondent answered yes to consider becoming a living kidney donor either for a family member, a friend or an unknown person |
| ***Explanatory variables*** |  |
| Sex_Female (Sex: Female) | Gender of respondent. Measures dummy variable, 1 if the responded is female and 0, otherwise |
| Age | Age of respondent in years. Measures as a continuous variable |
| CHILDNO | Number of children below 18 in household. Measured as a dummy variable:  CHILDNO1(Household with 1 child): 1 if there is one child in respondent’s household and 0, otherwise;  CHILDNO2 (Household with 2 children): 1 if there are 2 children in respondent’s household and 0, otherwise;  CHILDNO3 (Household with 3 children): 1 if there are 3 children in respondent’s household and 0, otherwise;  CHILDNO4 (Household with 4 children): 1 if there 4 children in respondent’s household and 0, otherwise;  CHILDNO5 (Household with 5 children): 1 if there are 5 children in respondent’s household and 0, otherwise;  CHILDNO6 (Household with more than 5 children): 1 if there are more than 5 children in household and 0, otherwise; |
| Ethnic origin (Ethnic_white (Ethnic origin: White)) | Ethnic background of respondent. Measures as a dummy variable, 1 if the respondent has a white ethnic background and 0, otherwise |
| Religion | Faith base of respondent. Measured as a dummy variable  Faith_Christianity (Religion: Christianity): 1 if the respondent is a Christian and 0, otherwise;  Faith_Islam (Religion: Islam): 1 if the respondent is a Muslim and 0, otherwise;  Faith_Hindusm (Religion: Hinduism): 1 if the respondent is a Hindu and 0, otherwise;  Faith_Sikhism (Religion: Sikhism): 1 if the respondent is a Sikh and 0, otherwise;  Faith_Buddhism (Religion: Buddhism): 1 if the respondent is a Buddhist and 0, otherwise;  Faith_Judaism (Religion: Judaism): I if the respondent is a Jew, and 0, otherwise  Faith_Other (Religion: Other): I if the respondent has other faith different from the aforementioned, and 0, otherwise |
| Occupation | Occupation of respondents. measured as a dummy variable.  Occupation_High_Professional (Occupation: High Professional): 1 if the respondent’s work belongs to high managerial administrative or professional category, and 0, otherwise  Occupation_Inter_Professional (Occupation: Intermediate Professional): 1 if the respondent’s work belongs to intermediate managerial administrative or professional category, and 0, otherwise  Occupation_Junior_Professional (Occupation: Junior Professional): 1 if the respondent’s work belongs to supervisor, clerical, junior managerial or professional category, and 0, otherwise  Occupation_Skilled_Worke (Occupation: Skilled Worker): 1 if the respondent’s work belongs to Skilled manual worker, e.g. mechanic category, and 0, otherwise  Occupation_Semiskilled_Worker (Occupation: Semiskilled Worker): 1 if the respondent’s work belongs to semi-skilled manual worker or unskilled manual worker category, and 0, otherwise  Occupation_Housewife_Husband (Occupation: Housewife Husband): 1 if the respondent is a housewife/househusband, and 0, otherwise  Occupation_Unemployed (Occupation: Unemployed): 1 if the respondent is unemployed, and 0, otherwise  Occupation_Student (Occupation: Student): 1 if the respondent is a student, and 0, otherwise |
| Publicity_aware (Awareness of organ donation publicity campaign) | Public awareness of organ donation publicity. Measured as a dummy variable.  1 of the respondent has ever seen, heard or read any news items, adverts, publicity or other types of information which focused on the subject of organ donation, and 0, otherwise |
| Support | Level of support for organ donation in principle. Measured as a dummy variable.  Support_OD (Support organ donation): 1 if the respondent supports organ donation is principle and 0, otherwise  Oppose_OD (Oppose organ donation): 1 if the respondent oppose organ donation is principle and 0, otherwise  Indifferent_OD (indifferent to organ donation): 1 if the respondent neither supports nor oppose organ donation in principle. |

Appendix 2: Mean/proportional estimate of covariates by quintile of predicted probability of living donation (Family sample)

|  | **Quintile1** | | **Quintile2** | | **Quinntile3** | | **Quintile4** | | **Quintile5** | |
| --- | --- | --- | --- | --- | --- | --- | --- | --- | --- | --- |
| **Covariates** | **Mean/**  **Proportion** | **Standard Error** | **Mean/**  **Proportion** | **Standard Error** | **Mean/**  **Proportion** | **Standard Error** | **Mean/**  **Proportion** | **Standard Error** | **Mean/**  **Proportion** | **Standard Error** |
| Sex: Female | 0.41 | 0.007 | 0.22 | 0.007 | 0.15 | 0.007 | 0.86 | 0.007 | 1.00 | 0.007 |
| Age | 40 | 0.318 | 41 | 0.318 | 49 | 0.318 | 42 | 0.318 | 44 | 0.318 |
| Household with one child | 0.14 | 0.006 | 0.17 | 0.006 | 0.14 | 0.006 | 0.14 | 0.006 | 0.11 | 0.006 |
| Household with two children | 0.13 | 0.006 | 0.15 | 0.006 | 0.10 | 0.006 | 0.11 | 0.006 | 0.09 | 0.006 |
| Household with three children | 0.04 | 0.003 | 0.05 | 0.003 | 0.02 | 0.003 | 0.03 | 0.003 | 0.02 | 0.003 |
| Household with four children | 0.02 | 0.002 | 0.01 | 0.002 | 0.01 | 0.002 | 0.01 | 0.002 | 0.00 | 0.002 |
| Household with five children | 0.01 | 0.001 | 0.00 | 0.001 | 0.00 | 0.001 | 0.00 | 0.001 | 0.00 | 0.001 |
| Ethnic origin: White | 0.69 | 0.007 | 0.73 | 0.007 | 0.86 | 0.007 | 0.85 | 0.007 | 0.99 | 0.007 |
| Religion: Christianity | 0.40 | 0.009 | 0.35 | 0.009 | 0.52 | 0.009 | 0.46 | 0.009 | 0.56 | 0.009 |
| Religion: Islam | 0.16 | 0.005 | 0.14 | 0.005 | 0.04 | 0.005 | 0.00 | 0.005 | 0.00 | 0.005 |
| Religion: Hinduism | 0.03 | 0.003 | 0.05 | 0.003 | 0.06 | 0.003 | 0.01 | 0.003 | 0.00 | 0.003 |
| Religion: Sikhism | 0.02 | 0.002 | 0.01 | 0.002 | 0.01 | 0.002 | 0.02 | 0.002 | 0.00 | 0.002 |
| Religion: Buddhism | 0.00 | 0.002 | 0.01 | 0.002 | 0.01 | 0.002 | 0.01 | 0.002 | 0.00 | 0.002 |
| Religion: Judaism | 0.01 | 0.001 | 0.01 | 0.001 | 0.01 | 0.001 | 0.01 | 0.001 | 0.01 | 0.001 |
| Occupation: High professional | 0.06 | 0.005 | 0.11 | 0.005 | 0.08 | 0.005 | 0.07 | 0.005 | 0.06 | 0.005 |
| Occupation: Intermediate professional | 0.18 | 0.008 | 0.29 | 0.008 | 0.24 | 0.008 | 0.19 | 0.008 | 0.26 | 0.008 |
| Occupation: Junior professional | 0.28 | 0.008 | 0.13 | 0.008 | 0.33 | 0.008 | 0.27 | 0.008 | 0.28 | 0.008 |
| Occupation: Skilled worker | 0.18 | 0.007 | 0.14 | 0.007 | 0.17 | 0.007 | 0.22 | 0.007 | 0.22 | 0.007 |
| Occupation: Semiskilled worker | 0.14 | 0.006 | 0.16 | 0.006 | 0.11 | 0.006 | 0.12 | 0.006 | 0.14 | 0.006 |
| Occupation: Housewife husband | 0.03 | 0.003 | 0.03 | 0.003 | 0.01 | 0.003 | 0.03 | 0.003 | 0.03 | 0.003 |
| Occupation: Unemployed | 0.10 | 0.004 | 0.11 | 0.004 | 0.04 | 0.004 | 0.06 | 0.004 | 0.00 | 0.004 |
| Occupation: Student | 0.05 | 0.003 | 0.04 | 0.003 | 0.02 | 0.003 | 0.05 | 0.003 | 0.00 | 0.003 |
| Awareness of organ donation publicity | 0.30 | 0.009 | 0.39 | 0.009 | 0.45 | 0.009 | 0.45 | 0.009 | 0.34 | 0.009 |
| Support organ donation | 0.00 | 0.003 | 0.84 | 0.003 | 1.00 | 0.003 | 1.00 | 0.003 | 1.00 | 0.003 |
| Oppose organ donation | 0.20 | 0.003 | 0.02 | 0.003 | 0.00 | 0.003 | 0.00 | 0.003 | 0.00 | 0.003 |

Appendix 3: Mean/proportional estimate of covariates by quintile of predicted probability of living donation (Friend sample)

|  | **Quintile1** |  | **Quintile2** |  | **Quinntile3** |  | **Quintile4** |  | **Quintile5** |  |
| --- | --- | --- | --- | --- | --- | --- | --- | --- | --- | --- |
| **Covariates** | **Mean/**  **Proportion** | **Standard Error** | **Mean/**  **Proportion** | **Standard Error** | **Mean/**  **Proportion** | **Standard Error** | **Mean/**  **Proportion** | **Standard Error** | **Mean/**  **Proportion** | **Standard Error** |
| Sex: Female | 0.50 | 0.009 | 0.47 | 0.009 | 0.48 | 0.009 | 0.56 | 0.009 | 0.63 | 0.009 |
| Age | 44 | 0.249 | 57 | 0.249 | 52 | 0.249 | 40 | 0.249 | 25 | 0.249 |
| Household with one child | 0.11 | 0.006 | 0.06 | 0.006 | 0.12 | 0.006 | 0.28 | 0.006 | 0.13 | 0.006 |
| Household with two children | 0.10 | 0.006 | 0.11 | 0.006 | 0.11 | 0.006 | 0.19 | 0.006 | 0.06 | 0.006 |
| Household with three children | 0.04 | 0.003 | 0.01 | 0.003 | 0.03 | 0.003 | 0.04 | 0.003 | 0.04 | 0.003 |
| Household with four children | 0.01 | 0.002 | 0.01 | 0.002 | 0.01 | 0.002 | 0.01 | 0.002 | 0.01 | 0.002 |
| Household with five children | 0.01 | 0.001 | 0.00 | 0.001 | 0.00 | 0.001 | 0.00 | 0.001 | 0.00 | 0.001 |
| Ethnic origin: White | 0.74 | 0.007 | 0.85 | 0.007 | 0.81 | 0.007 | 0.79 | 0.007 | 0.91 | 0.007 |
| Religion: Christianity | 0.43 | 0.009 | 0.53 | 0.009 | 0.47 | 0.009 | 0.54 | 0.009 | 0.33 | 0.009 |
| Religion: Islam | 0.12 | 0.005 | 0.07 | 0.005 | 0.09 | 0.005 | 0.04 | 0.005 | 0.02 | 0.005 |
| Religion: Hinduism | 0.03 | 0.003 | 0.02 | 0.003 | 0.03 | 0.003 | 0.05 | 0.003 | 0.01 | 0.003 |
| Religion: Sikhism | 0.02 | 0.002 | 0.01 | 0.002 | 0.01 | 0.002 | 0.02 | 0.002 | 0.01 | 0.002 |
| Religion: Buddhism | 0.01 | 0.002 | 0.01 | 0.002 | 0.01 | 0.002 | 0.01 | 0.002 | 0.01 | 0.002 |
| Religion: Judaism | 0.01 | 0.001 | 0.01 | 0.001 | 0.01 | 0.001 | 0.01 | 0.001 | 0.00 | 0.001 |
| Occupation: High professional | 0.04 | 0.005 | 0.07 | 0.005 | 0.07 | 0.005 | 0.09 | 0.005 | 0.10 | 0.005 |
| Occupation: Intermediate professional | 0.16 | 0.008 | 0.29 | 0.008 | 0.20 | 0.008 | 0.26 | 0.008 | 0.25 | 0.008 |
| Occupation: Junior professional | 0.26 | 0.008 | 0.25 | 0.008 | 0.28 | 0.008 | 0.27 | 0.008 | 0.24 | 0.008 |
| Occupation: Skilled worker | 0.20 | 0.007 | 0.17 | 0.007 | 0.20 | 0.007 | 0.19 | 0.007 | 0.17 | 0.007 |
| Occupation: Semiskilled worker | 0.18 | 0.006 | 0.13 | 0.006 | 0.17 | 0.006 | 0.10 | 0.006 | 0.10 | 0.006 |
| Occupation: Housewife husband | 0.03 | 0.003 | 0.02 | 0.003 | 0.02 | 0.003 | 0.03 | 0.003 | 0.02 | 0.003 |
| Occupation: Unemployed | 0.10 | 0.004 | 0.06 | 0.004 | 0.06 | 0.004 | 0.04 | 0.004 | 0.04 | 0.004 |
| Occupation: Student | 0.04 | 0.003 | 0.01 | 0.003 | 0.01 | 0.003 | 0.02 | 0.003 | 0.08 | 0.003 |
| Awareness of organ donation publicity | 0.23 | 0.009 | 0.40 | 0.009 | 0.34 | 0.009 | 0.48 | 0.009 | 0.49 | 0.009 |
| Support organ donation | 0.00 | 0.003 | 0.84 | 0.003 | 1.00 | 0.003 | 1.00 | 0.003 | 1.00 | 0.003 |
| Oppose organ donation | 0.12 | 0.004 | 0.09 | 0.004 | 0.00 | 0.004 | 0.00 | 0.004 | 0.00 | 0.004 |

Appendix 4: Mean/proportional estimate of covariates by quintile of predicted probability of living donation (Unknown person sample)

|  | **Quintile1** | | **Quintile2** | | **Quinntile3** | | **Quintile4** | | **Quintile5** | |
| --- | --- | --- | --- | --- | --- | --- | --- | --- | --- | --- |
| **Covariates** | **Mean/**  **Proportion** | **Standard Error** | **Mean/**  **Proportion** | **Standard Error** | **Mean/**  **Proportion** | **Standard Error** | **Mean/**  **Proportion** | **Standard Error** | **Mean/**  **Proportion** | **Standard Error** |
| Sex: Female | 0.52 | 0.009 | 0.55 | 0.009 | 0.47 | 0.009 | 0.49 | 0.009 | 0.60 | 0.009 |
| Age | 46 | 0.298 | 50 | 0.298 | 48 | 0.298 | 40 | 0.298 | 31 | 0.298 |
| Household with one child | 0.09 | 0.006 | 0.07 | 0.006 | 0.15 | 0.006 | 0.21 | 0.006 | 0.18 | 0.006 |
| Household with two children | 0.08 | 0.006 | 0.12 | 0.006 | 0.15 | 0.006 | 0.14 | 0.006 | 0.09 | 0.006 |
| Household with three children | 0.04 | 0.003 | 0.02 | 0.003 | 0.03 | 0.003 | 0.05 | 0.003 | 0.03 | 0.003 |
| Household with four children | 0.01 | 0.002 | 0.01 | 0.002 | 0.01 | 0.002 | 0.01 | 0.002 | 0.01 | 0.002 |
| Household with five children | 0.00 | 0.001 | 0.01 | 0.001 | 0.00 | 0.001 | 0.00 | 0.001 | 0.00 | 0.001 |
| Ethnic origin: White | 0.76 | 0.007 | 0.92 | 0.007 | 0.93 | 0.007 | 0.77 | 0.007 | 0.74 | 0.007 |
| Religion: Christianity | 0.39 | 0.009 | 0.52 | 0.009 | 0.54 | 0.009 | 0.43 | 0.009 | 0.42 | 0.009 |
| Religion: Islam | 0.13 | 0.005 | 0.03 | 0.005 | 0.02 | 0.005 | 0.08 | 0.005 | 0.07 | 0.005 |
| Religion: Hinduism | 0.02 | 0.003 | 0.01 | 0.003 | 0.01 | 0.003 | 0.05 | 0.003 | 0.05 | 0.003 |
| Religion: Sikhism | 0.02 | 0.002 | 0.00 | 0.002 | 0.00 | 0.002 | 0.01 | 0.002 | 0.02 | 0.002 |
| Religion: Buddhism | 0.01 | 0.002 | 0.01 | 0.002 | 0.01 | 0.002 | 0.01 | 0.002 | 0.01 | 0.002 |
| Religion: Judaism | 0.01 | 0.001 | 0.01 | 0.001 | 0.01 | 0.001 | 0.01 | 0.001 | 0.00 | 0.001 |
| Occupation: High professional | 0.04 | 0.005 | 0.04 | 0.005 | 0.08 | 0.005 | 0.12 | 0.005 | 0.10 | 0.005 |
| Occupation: Intermediate professional | 0.20 | 0.008 | 0.28 | 0.008 | 0.26 | 0.008 | 0.20 | 0.008 | 0.22 | 0.008 |
| Occupation: Junior professional | 0.26 | 0.008 | 0.36 | 0.008 | 0.26 | 0.008 | 0.23 | 0.008 | 0.16 | 0.008 |
| Occupation: Skilled worker | 0.19 | 0.007 | 0.17 | 0.007 | 0.20 | 0.007 | 0.17 | 0.007 | 0.20 | 0.007 |
| Occupation: Semiskilled worker | 0.16 | 0.006 | 0.09 | 0.006 | 0.14 | 0.006 | 0.16 | 0.006 | 0.11 | 0.006 |
| Occupation: Housewife husband | 0.03 | 0.003 | 0.02 | 0.003 | 0.02 | 0.003 | 0.03 | 0.003 | 0.02 | 0.003 |
| Occupation: Unemployed | 0.09 | 0.004 | 0.02 | 0.004 | 0.03 | 0.004 | 0.06 | 0.004 | 0.10 | 0.004 |
| Occupation: Student | 0.03 | 0.003 | 0.02 | 0.003 | 0.01 | 0.003 | 0.03 | 0.003 | 0.08 | 0.003 |
| Awareness of organ donation publicity | 0.17 | 0.009 | 0.31 | 0.009 | 0.35 | 0.009 | 0.48 | 0.009 | 0.62 | 0.009 |
| Support organ donation | 0.08 | 0.004 | 0.84 | 0.004 | 0.96 | 0.004 | 0.97 | 0.004 | 1.00 | 0.004 |
| Oppose organ donation | 0.14 | 0.004 | 0.04 | 0.004 | 0.02 | 0.004 | 0.01 | 0.004 | 0.00 | 0.004 |
